# Supplementary material for: Perceptions and knowledge of breast cancer and breast self-examination among young adult women in southwest Ethiopia: Application of the health belief model
Source: PLoS One. 2022 Sep 21;17(9):e0274935. doi: 10.1371/journal.pone.0274935 (PMC9491534; doi:10.1371/journal.pone.0274935)
Supplement: Attachment — Submitted filename: PLos one- Perceptions and knowledge of breast cancer and breast self 2022.docx [file pone.0274935.s001.docx]

Perceptions and knowledge of breast cancer and breast self-examination among  young adult Ethiopian women: application of the health belief model .

**REVIEW COMMENTS**

The study was carried out to assess the knowledge and perceptions of young adult women in Southwest Ethiopia about breast cancer and breast self-examination (BSE). This is a good manuscript with very robust methods. The methodology has been comprehensibly and logically described for reproducibility. The write up and presentation good, however, some concerns need to be addressed. I recommend a major revision

**Major Revision**

**Abstract**

**Methods**

Line 24 State the community used

begin with…. A total of 392

Line 31 with a mean of 21.25 years (sd = 1.32)…. rewrite as Mean (±SD) ie.

21.25( ±1.32)

Line 35 “on occasion” should be replaced with…occasionally.

**Conclusion**

BC should be defined on first use

**Introduction**

Line 53 delete “lying”

Line 55 is the sentence in reference to Ethiopia? If otherwise can author back up with references?

Line 61 “Young women's cancers are mostly diagnosed at an advanced stage” Could be rewritten as….. Young women with cancer are often diagnosed with advanced stage breast cancer.

Line 72 these references does not speak BSE in Ethiopia but rather other African countries.. Could a reference to that effect be included? Or sentence rephrased?

**Materials and Methods**

Line 89 period design…? What is that exactly

Line 99 study design: Information on study design is quite scanty I recommend a beef up. Or can be merged with study area… as study area and study design.

Line 103 were the young adults purposively selected? Please state so.

Line 125 The data were collected by 25 trained health extension workers under the supervision of five health officers and nurses…..Considering this information, I find it intriguing that this manuscript is a sole authorship one. Any comments on this?

Line 126… “that were closed households or had no participants were visited three times”. Should be rephrased sentence seem incomplete.

Line 133 “signals” ... change to “signs”

Line 142 BSE forms?

Line 188 the appropriate consent for participation should be a written informed consent.

**Results**

**Line 193** Mean And SD data should be presented in standard formats

**write as…. Mean (±SD)**

Table 1 remove the year in the title.

If the questionnaire was self-administered, how was the 2.4% illiterate population assessed using the questionnaire? This must be elaborated in the methods.

Table 1 A 7.8% of married young adult in the study aea is worth a mention in the discussion.

Mean And SD data should be presented in standard formats through the manuscript

Percent should be written as Percent (%) to avoid the use of the symbol through the table

Academic status could be replaced with Educational Background.

The inclusion of the year in the table and figure titles should be revised.

Table 2 Percent should be accompanied by its Symbol.

Line 240.. title is not reflective of the information provided

**Discussion**

Line 273.. only one study is cited.

Line 275.. what is accounting for the poor knowledge in your study area?

Line 267 why so? Any suggestions to improve this outcome. That should a more precise focus of the discussion. Not merely stating what the data says.

Line 283 what is accounting for this low/poor knowledge BSE in south Ethiopia or your study population. Explain

Use consistent decimal points for all the percentage sated in the tables and discussion

Line 311 it is .. rather than it’s..

Line 317 “Confidence is supposed to come after knowledge”. Can be rewritten as….. “Knowledge builds confidence”

Line 323 what some of these barriers… as they are not mentioned clearly in the write up

Line 328 it is .. rather than it’s..

Line 332 I think that….. “The possible limitation of this work is that perceptions and behavioral practices of other breast cancer screening methods, such as clinical breast examination and mammography, were not assessed”. …. Is not the scope of the study and thus does not constitute a study limitation but may be a recommendation for additional studies. Reconsider!

**Conclusion**

Line 339 may not be necessary as part of the conclusion. Conclusion should be precise, concise, straight to the point.
