# Supplementary material for: Perceptions and knowledge of breast cancer and breast self-examination among young adult women in southwest Ethiopia: Application of the health belief model
Source: PLoS One. 2022 Sep 21;17(9):e0274935. doi: 10.1371/journal.pone.0274935 (PMC9491534; doi:10.1371/journal.pone.0274935)
Supplement: Attachment — Submitted filename: Response to Reviewers.docx [file pone.0274935.s002.docx]

# June 21, 2022

From: Authors

To: PLOS ONE editorials

**Subject: Response to reviewers**

Dear Editor and reviewers:

I would like to thank the editors and reviewers for their time, careful reading, and thorough and helpful remarks and questions about my manuscript, as well as the opportunity to improve and resubmit it.

I believe that my revised paper has substantially improved as a result of the reviewers’ remarks. I am glad to submit the revised research manuscript for consideration in PLOS ONE with a suggested small title modification.

I responded to each reviewer's questions, comments, and suggested revisions one by one. I used the same letter to write my responses after copying the review decision from the editorial manager's submission menu. Editors' and reviewers' questions and comments are *italicized*, and my responses to the reviewers' comments are presented in plain text immediately below them. All of the modifications I made in the revised manuscript were referenced by page and line numbers. Please, follow my responses in a yellow mark to every comment or question in a green mark.

I uploaded the data set in figshare public data repository ( Doi=10.6084/m9.figshare.20103206

URLs= <https://doi.org/10.6084/m9.figshare.20103206>)

Once again, thank you for your time and attention to my paper***,*** and I eagerly await your response.

Sincerely!

Kenzudin Assfa Mossa (corresponding author)

Wolkite University

[kenzaheri@gmail.com](mailto:kenzaheri@gmail.com%20)

+251923702054

PONE-D-22-07638

Perceptions and knowledge of breast cancer and breast self-examination among young adult Ethiopian women: application of the health belief model

PLOS ONE

Dear Dr. Assfa,

Thank you for submitting your manuscript to PLOS ONE. After careful consideration, we feel that it has merit but does not fully meet PLOS ONE’s publication criteria as it currently stands. Therefore, we invite you to submit a revised version of the manuscript that addresses the points raised during the review process.

Please submit your revised manuscript by Jun 23 2022 11:59PM. If you will need more time than this to complete your revisions, please reply to this message or contact the journal office at [plosone@plos.org](mailto:plosone@plos.org). When you're ready to submit your revision, log on to <https://www.editorialmanager.com/pone/> and select the 'Submissions Needing Revision' folder to locate your manuscript file.

Please include the following items when submitting your revised manuscript:

- A rebuttal letter that responds to each point raised by the academic editor and reviewer(s). You should upload this letter as a separate file labeled 'Response to Reviewers'.
- A marked-up copy of your manuscript that highlights changes made to the original version. You should upload this as a separate file labeled 'Revised Manuscript with Track Changes'.
- An unmarked version of your revised paper without tracked changes. You should upload this as a separate file labeled 'Manuscript'.

If you would like to make changes to your financial disclosure, please include your updated statement in your cover letter. Guidelines for resubmitting your figure files are available below the reviewer comments at the end of this letter.

If applicable, we recommend that you deposit your laboratory protocols in [protocols.io](http://protocols.io/) to enhance the reproducibility of your results. Protocols.io assigns your protocol its own identifier (DOI) so that it can be cited independently in the future.

For instructions see: <https://journals.plos.org/plosone/s/submission-guidelines#loc-laboratory-protocols>. Additionally, PLOS ONE offers an option for publishing peer-reviewed Lab Protocol articles, which describe protocols hosted on [protocols.io](http://protocols.io/). Read more information on sharing protocolsat[https://plos.org/protocols?utm_medium=editorial email&utm_source=authorletters&utm_campaign=protocols](https://plos.org/protocols?utm_medium=editorial%20email&utm_source=authorletters&utm_campaign=protocols).

We look forward to receiving your revised manuscript.

Kind regards,

Alvaro Galli

Academic Editor

PLOS ONE

 Journal Requirements:

When submitting your revision, we need you to address these additional requirements.

Comments :*1. Please ensure that your manuscript meets PLOS ONE's style requirements, including those for file naming. The PLOS ONE style templates can be found at* [*https://journals.plos.org/plosone/s/file?id=wjVg/PLOSOne_formatting_sample_main_body.pdf*](https://journals.plos.org/plosone/s/file?id=wjVg/PLOSOne_formatting_sample_main_body.pdf) *&*[*https://journals.plos.org/plosone/s/file?id=ba62/PLOSOne_formatting_sample_title_authors_affiliations.pdf*](https://journals.plos.org/plosone/s/file?id=ba62/PLOSOne_formatting_sample_title_authors_affiliations.pdf)

Responses#: Thank you for your comment. I formatted the manuscript in accordance with PLOS ONE's style guidelines and template.

Comments: *2. you indicated that you had ethical approval for your study. In your Methods section, please ensure you have also stated whether you obtained consent from parents or guardians of the minors included in the study or whether the research ethics committee or IRB specifically waived the need for their consent.*

Responses#: Your suggestion is much appreciated. Participants in this study, however, were young adult women aged 20 to 24, who had reached the legal age to consent to participate in the study independently under Ethiopian law. As a result, no parental or guardian consent was requested.

*3. In your Data Availability statement, you have not specified where the minimal data set underlying the results described in your manuscript can be found. PLOS defines a study's minimal data set as the underlying data used to reach the conclusions drawn in the manuscript and any additional data required to replicate the reported study findings in their entirety. All PLOS journals require that the minimal data set be made fully available. For more information about our data policy, please see* [*http://journals.plos.org/plosone/s/data-availability*](http://journals.plos.org/plosone/s/data-availability)*.*

*Comments: Upon re-submitting your revised manuscript, please upload your study’s minimal underlying data set as either Supporting Information files or to a stable, public repository and include the relevant URLs, DOIs, or accession numbers within your revised cover letter. For a list of acceptable repositories, please see* [*http://journals.plos.org/plosone/s/data-availability#loc-recommended-repositories*](http://journals.plos.org/plosone/s/data-availability#loc-recommended-repositories)*. Any potentially identifying patient information must be fully anonymized.*

Responses#: Thank you very much; I uploaded my data availability statement so that the minimum of data set is fully available in figshare public data repository (Doi=10.6084/m9.figshare.20103206 ,URLs= <https://doi.org/10.6084/m9.figshare.20103206>)

*Important: If there are ethical or legal restrictions to sharing your data publicly, please explain these restrictions in detail. Please see our guidelines for more information on what we consider unacceptable restrictions to publicly sharing data:* [*http://journals.plos.org/plosone/s/data-availability#loc-unacceptable-data-access-restrictions*](http://journals.plos.org/plosone/s/data-availability#loc-unacceptable-data-access-restrictions)*. Note that it is not acceptable for the authors to be the sole named individuals responsible for ensuring data access. We will update your Data Availability statement to reflect the information you provide in your cover letter.*

[Note: HTML markup is below. Please do not edit.]

Reviewers' comments:

Reviewer's Responses to Questions

**Comments to the Author**

1. Is the manuscript technically sound, and do the data support the conclusions?

The manuscript must describe a technically sound piece of scientific research with data that supports the conclusions. Experiments must have been conducted rigorously, with appropriate controls, replication, and sample sizes. The conclusions must be drawn appropriately based on the data presented.

Reviewer #1: Partly

Reviewer #2: Yes

2. Has the statistical analysis been performed appropriately and rigorously?

Reviewer #1: Yes

Reviewer #2: Yes

*Comments: 3. Have the authors made all data underlying the findings in their manuscript fully available?

The* [*PLOS Data policy*](http://www.plosone.org/static/policies.action#sharing) *requires authors to make all data underlying the findings described in their manuscript fully available without restriction, with rare exception (please refer to the Data Availability Statement in the manuscript PDF file). The data should be provided as part of the manuscript or its supporting information, or deposited to a public repository. For example, in addition to summary statistics, the data points behind means, medians and variance measures should be available. If there are restrictions on publicly sharing data—e.g. participant privacy or use of data from a third party—those must be specified.*

*Reviewer #1: No*

*Reviewer #2: No*

Responses#: Thank you very much; I updated my data availability statement so that the minimum of data set is fully available in figshare public data repository (Doi=10.6084/m9.figshare.20103206 ,URLs= <https://doi.org/10.6084/m9.figshare.20103206>) .

4. Is the manuscript presented in an intelligible fashion and written in standard English?

PLOS ONE does not copyedit accepted manuscripts, so the language in submitted articles must be clear, correct, and unambiguous. Any typographical or grammatical errors should be corrected at revision, so please note any specific errors here.

Reviewer #1: Yes

Reviewer #2: Yes

5. Review Comments to the Author

Please use the space provided to explain your answers to the questions above. You may also include additional comments for the author, including concerns about dual publication, research ethics, or publication ethics. (Please upload your review as an attachment if it exceeds 20,000 characters)

Reviewer #1: Perceptions and knowledge of breast cancer and breast self-examination among young adult Ethiopian women: application of the health belief model

REVIEW COMMENTS

The study was carried out to assess the knowledge and perceptions of young adult women in Southwest Ethiopia about breast cancer and breast self-examination (BSE). This is a good manuscript with very robust methods. The methodology has been comprehensibly and logically described for reproducibility. The write up and presentation good, however, some concerns need to be addressed. I recommend a major revision

Major Revision

Abstract
Methods
*comments: Line 24 State the community used begin with…. A total of 392*

Responses: I'm grateful to the reviewer who brought this to my attention. I have revised it. The changes made are indicated in track changed document. The changes are also found on the revised manuscript document [pages2, lines 24].

*comments:Line 31 with a mean of 21.25 years (sd = 1.32)…. rewrite as Mean (±SD) ie.
21.25( ±1.32)*

Responses I agree and have updated line 31. The new sentence reads as follows “The respondents' ages ranged from 20 to 24, with a mean of 21.25 (*±* 1.32) years.” The changes made are indicated in track changed document. The changes are also found on the revised manuscript document [pages2, lines 30].

*comments:Line 35 “on occasion” should be replaced with…occasionally*

Response #: I agree and have changed “on occasion” to “occasionally”. The changes made are indicated in track changed document. The changes are also found on the revised manuscript document [pages2, lines 34].

*Conclusion
comments: BC should be defined on first use*

Response #: This is a valid observation. “BC” has been replaced with “breast cancer”. The changes made are indicated in track changed document. The changes are also found on the revised manuscript document [pages2, lines 39].

*Introduction
comments: Line 53 delete “lying”*

Response #: I agree and have removed the term "lying" from the sentence.

*comments: Line 55 is the sentence in reference to Ethiopia? If otherwise can author back up with references?*

Response #: Thank you for sharing your thoughts. I've provided references to back it up. The changes made are indicated in track changed document. The changes are also found on the revised manuscript document [pages3, lines 52].

*comments: Line 61 “Young women's cancers are mostly diagnosed at an advanced stage” Could be rewritten as….. Young women with cancer are often diagnosed with advanced stage breast cancer.*

Response #: I agree with you, and I've rewritten it as follows: “Young women with cancer are often diagnosed with advanced stage breast cancer.” The changes made are indicated in track changed document. The changes are also found on the revised manuscript document [pages3, lines 58].

*comments:Line 72 these references does not speak BSE in Ethiopia but rather other African countries. Could a reference to that effect be included? Or sentence rephrased?*

Response #: This observation is correct. I have included a reference to Ethiopia’s BSE problem. The changes made are indicated in track changed document. The changes are also found on the revised manuscript document [pages4, lines 73].

*Materials and Methods*

*comments:Line 89 period design…? What is that exactly*

Response #: I am sorry for the error. “Study area, study period, and study design" was supposed to be the meaning. I've fixed the problem, and it's now written as “study area and study design”. The changes made are indicated in track changed document. The changes are also found on the revised manuscript document [pages5, lines 95].

*comments:Line 99 study design: Information on study design is quite scanty I recommend a beef up. Or can be merged with study area… as study area and study design.*

Response #: I welcome your insightful suggestion, and it has been combined with the study area and study design.

*comments: Line 103 were the young adults purposively selected? Please state so.*

Response #: Thank you for alerting me to this; however, I do not believe it is better to state so. Because I used a probability sampling approach rather than a purposive approach in the recruitment process. I hope you will keep me updated on your second version revision process if I don't get your view point right.

*comments: Line 125 The data were collected by 25 trained health extension workers under the supervision of five health officers and nurses….Considering this information, I find it intriguing that this manuscript is a sole authorship one. Any comments on this?*

Response #: Thank you for drawing my attention to this. I think it is important to briefly describe my data collectors who are the Health Extension Workers (HEW). According to Ethiopia's present health-care delivery system, each kebele (a smaller administrative unit) is allotted one or two HEW. They are dominantly female who expected to be well-versed in the community's culture and to be native speakers of the local language. They provide 16 different packages of basic health services. They also do a home visit and organize health and associated data, which is then documented in an organized manner in each family folder in their health post. In general, HEWs are valuable information sources with a broad spectrum of knowledge about their society. That's why I used them to gather my data. For each selected kebele, I used one HEW as responsible data collector to gather data in their respective kebele only without sending them to other kebele to keep their routine tasks on track as usual. That is why the data collectors’ number seems to be higher.

*comments:Line 126… “that were closed households or had no participants were visited three times”. Should be rephrased sentence seem incomplete.*

Response #: Thank you so much for your good insight, I have rephrased the sentence and the new sentence reads as follows: “Households that were either closed or lacked a respondent were visited three times.” The changes made are indicated in track changed document. The changes are also found on the revised manuscript document [pages7, lines 131].

*comments: Line 133 “signals” ... change to “signs”*

Response #: I agree with you, I have changed “signals” to “signs”. The changes made are indicated in track changed document. The changes are also found on the revised manuscript document [pages7, lines 139].

*Comments: Line 142 BSE forms?*

Response #: My apologies for using unfamiliar terminology, it was to mean a tool to measure BSE. I have rewritten it as “BSE knowledge measurement” The changes made are indicated in track changed document. The changes are also found on the revised manuscript document [pages7, lines 148].

*comments:Line 188 the appropriate consent for participation should be a written informed consent.*

Response #: I agree with you that written informed consent is preferable. Even if I use verbal consent, data collectors fully explain the study's purpose, anonymity, confidentiality, and voluntariness of participation before obtaining participants' consent or collecting data.

*Results
comments: Line 193 Mean and SD data should be presented in standard formats write as…. Mean (±SD)*

Response #: Thank you for your comment, I have revised and the result has been amended and the new sentence reads as follows: “The participants' ages ranged from 20 to 24, with a mean of 21.25(±1.32) years.” The changes made are indicated in track changed document. The changes are also found on the revised manuscript document [pages10, lines 199].

*comments:Table 1 remove the year in the title.*

Response #: I agree with you, I have removed the ‘year” in the title of table 1 [pages10, lines 205].

*comments:If the questionnaire was self-administered, how was the 2.4% illiterate population assessed using the questionnaire? This must be elaborated in the methods.*

Response #: I appreciate the reviewer bringing this to my attention. In response to reviewers' suggestions, I revised and added additional elaboration to the data collection procedure. The changes made are indicated in track changed document. The changes are also found on the revised manuscript document [pages7, lines 134-138].

*comments:Table 1 A 7.8% of married young adult in the study area is worth a mention in the discussion.*

Response #: Thank you for taking the time to leave such an insightful comment. In the discussion section of the manuscript, I mentioned the marital status of the study participants. The modifications made are noted in the document's track changed section. The changes are reflected in the amended manuscript as well [pages 19, lines 316-323].

*comments: Mean And SD data should be presented in standard formats through the manuscript*

Response #: I agree with you and I have rewritten the Mean and SD in standard format [page 10, line 199]

*comments: Percent should be written as Percent (%) to avoid the use of the symbol through the table*

Response #: I agree with you and I have written the Percent as Percent (%) [ table 1 column 4 row1] and the symbols % in the table 1 have been removed [ table 1 column 4].

*comments:Academic status could be replaced with Educational Background.*

Response #: Thank you for the feedback I have replaced “Academic status” by “Educational Background” [table 1 column 1 row 4]

*comments: The inclusion of the year in the table and figure titles should be revised.*

Response #: Thank you for the suggestion, I have revised the year in all table and fig title [page 10, line 205] [page 11, line 216,217] [page 13, line 228,231] [page 14, line 238] [page 15, line 248] and [page 16, line 261,262].

*comments:* *Table 2 Percent should be accompanied by its Symbol.*

Response #: Thank you, Percent in able 2 has written as Percent (%) [page 11, table 2 column 4]

*comments: Line 240.. title is not reflective of the information provided*

Response #: Thank you for your feedback; if my understanding of your concern about line 240 is correct, it is a subheading given for young adult women's perception towards breast cancer and BSE, not the title of the table (table-4) above it. Young adult women's perceptions of breast cancer and BSE have been described under this subheading, which includes their perceived (susceptibility, severity, benefit, barriers, threat, outcome expectation or net benefit of BSE, and perceived confidence or self-efficacy). I have slightly modified the subheading. The changes made are indicated in track changed document. The changes are also found on the revised manuscript document [pages 15, lines 240].

*Discussion
comments: Line 273. only one study is cited.*

Response #: I agree and have updated with two additional references. The changes made are indicated in track changed document. The changes are also found on the revised manuscript document [pages 18, lines 284].

*comments: Line 275.. what is accounting for the poor knowledge in your study area?*

Response #: Thank you for the question. It is possible that this is due to the fact that cancer receives less attention [page 17,278-279]

*comments: Line 267 why so? Any suggestions to improve this outcome. That should a more precise focus of the discussion. Not merely stating what the data says.*

Response #: Thank you for taking the time to leave such an insightful comment. I've included possible contributing factors as well as recommendations for raising young women's awareness of breast health. The changes are added in the track changed manuscript document. They are also found on the revised manuscript document on [page 17, lines 278-280].

*comments: Line 283 what is accounting for this low/poor knowledge BSE in south Ethiopia or your study population. Explain*

Response #: Thank you for your valuable insight. I have added and discussed the possible contributing factors for respondents (young adult women) poor knowledge in my study area. The changes are added in the track changed manuscript document. They are also found on the revised manuscript document on [page 18, lines 300-303].

*comments: Use consistent decimal points for all the percentage sated in the tables and discussion*

Response #: I appreciate and thank you for your thoughtful remarks. I agree with your point of view. All fractions have been rounded to the nearest two decimal places for all the percentages sated in the paper. The changes are made in the track changed manuscript document

*comments:Line 311 it is .. rather than it’s.*

Response #: I have replaced the word “it’s” by “it is” [page 20, lines 333].

*comments:Line 317 “Confidence is supposed to come after knowledge”. Can be rewritten as….. “Knowledge builds confidence”*

Response #: I accepted your suggestion and have rewritten as “Knowledge builds confidence” The changes are added in the track changed manuscript document. They are also found on the revised/amended manuscript document on [page 20, lines 339-340].

*comments: Line 323 what some of these barriers… as they are not mentioned clearly in the write up*

Response #: Thank you for your question and suggestion. In this study perceived barriers of BSE were measured using 7 items stated as: (1) BSE is embarrassing to me, (2) BSE takes too much time, (3) It is hard to remember to do breast examination, (4) I am afraid I would not be able to do breast self-exams,(5) I don't have enough privacy to do breast examination, (6) BSE is not necessary if you have a breast exam by a healthcare provider and (7) I have other problems more important than doing BSE. Overall, lack of knowledge, skill and privacy to perform BSE are some of the barriers. Apart from that, believing that BSE embracing, and time-consuming activity which is also related with lower knowledge, giving less priority for them practices compared to another problem which they believe important than BSE. The changes are added in the track changed manuscript document. They are also found on the revised/amended manuscript document on [page 20, Line 345-346].

*comments:Line 328 it is .. rather than it’s..*

Response #: Thank you, I have replaced the word “it’s” by “it is” [ page 21, Line 350].

*comments: Line 332 I think that….. “The possible limitation of this work is that perceptions and behavioral practices of other breast cancer screening methods, such as clinical breast examination and mammography, were not assessed”. …. Is not the scope of the study and thus does not constitute a study limitation but may be a recommendation for additional studies. Do Reconsider!*

Response #: Thank you for your valuable suggestion. The statement “The possible limitation of this work is that perceptions and behavioral practices of other breast cancer screening methods, such as clinical breast examination and mammography, were not assessed” has been deleted. The changes are added in the track changed manuscript document.

*Conclusion
comments: Line 339 may not be necessary as part of the conclusion. Conclusion should be precise, concise, straight to the point.*

Response #: The statement in conclusion “In this study, health beliefs and reported breast self-examination practices of young adult women were assessed.” has been removed as suggested by reviewer. The changes are added in the track changed manuscript document.

Reviewer #2:

*comments:1. It is not clear why only women 20-24 were included in the study while older women are also eligible for BSE. This raises a question in the significance of the study? Otherwise, the findings need to be put in context and discussed accordingly*

Response #: I appreciated and thank you for your valuable insight. I have provided additional points to answer why only young adult women (20-24 years old) were included in my study. The change has been highlighted in the track changed manuscript documented. It is found on [page 3 lines number 58-61], [page 4, line number 74-76] and [page 4, line number 81-83] in the revised manuscript document.

*comments:2. The title of the study seems as if the study is generalizable to Ethiopian women but only included women in one town. This is misleading and need to be corrected to mention the study locality in the title*

Response #: Thank you for your concern and question. I agree with you, thus I've slightly modified the title to look like: “Perceptions and knowledge of breast cancer and breast self-examination among young adult women in South west Ethiopia: application of the health belief model”. The change has been highlighted in the track changed manuscript documented and it is found on [page 1, lines number 2] in the revised manuscript document.

*comments:3. The samples size assumption of 20% is taken not from Ethiopian or African women. The SS is difficult to ensure adequacy. In such cases 50% should have been taken. In addition, no justification given why design-effect if only 1.5 was taken while ti could have been 2. All these raise concerns on the adequacy of sample size.*

Response #: I appreciate the reviewer who brought this to my attention. I totally agree that the safest choice for obtaining the largest sample size is to use a population proportion of 50%. When it comes to the design effect, researchers adopt a default value of 1.5 to 2.0 when determining sample size because the design effect (*deff*) is typically regarded as unknown before to a survey. To account for the expense, workload, and time required to collect data from a geographically scattered study population, I utilized the design effect 1.5 and p=0.203 in my sample.

*comments:4. The health belief model tool is a generic one. There is no mention of reliability and validity test for Ethiopian population. How can we rely in this measurement?*

Response #: I agree with you and thank you for your concern. I've reported the Cronbach alpha as a measure of consistency[page 8, L 163-164]. An expert panel was consulted to ensure face validity when the tool's validity was examined.

*comments:5. The data in Table 6 (association test) are not fully presented for most cells. In addition, data only for those significantly associated is included. This is not appropriate. All variables irrespective of status if association were supposed to be presented.*

Response #: Thank you for taking the time to provide me with your valuable input. A change has been made to the table. If my understanding is correct, the table contained all variables, independent of their significant status. Continuous independent variables were denoted by the letter “a” in superscript style, and their cells remained vacant. The change has been highlighted in the track changed manuscript documented and it is found on [page 17, line number 262-263] and [table 6 column 1 row 9-11] in the revised manuscript document.

6. PLOS authors have the option to publish the peer review history of their article ([what does this mean?](https://journals.plos.org/plosone/s/editorial-and-peer-review-process#loc-peer-review-history)). If published, this will include your full peer review and any attached files.

If you choose “no”, your identity will remain anonymous but your review may still be made public.

**Do you want your identity to be public for this peer review?** For information about this choice, including consent withdrawal, please see our [Privacy Policy](https://www.plos.org/privacy-policy).

Reviewer #1: No

Reviewer #2: No

 [NOTE: If reviewer comments were submitted as an attachment file, they will be attached to this email and accessible via the submission site. Please log into your account, locate the manuscript record, and check for the action link "View Attachments". If this link does not appear, there are no attachment files.]

While revising your submission, please upload your figure files to the Preflight Analysis and Conversion Engine (PACE) digital diagnostic tool, <https://pacev2.apexcovantage.com/>. PACE helps ensure that figures meet PLOS requirements. To use PACE, you must first register as a user. Registration is free. Then, login and navigate to the UPLOAD tab, where you will find detailed instructions on how to use the tool. If you encounter any issues or have any questions when using PACE, please email PLOS at [figures@plos.org](mailto:figures@plos.org). Please note that Supporting Information files do not need this step.

Thank you

The Author
