# Supplementary material for: Perceptions and knowledge of breast cancer and breast self-examination among young adult women in southwest Ethiopia: Application of the health belief model
Source: PLoS One. 2022 Sep 21;17(9):e0274935. doi: 10.1371/journal.pone.0274935 (PMC9491534; doi:10.1371/journal.pone.0274935)
Supplement: Attachment — Submitted filename: Response to Reviewers.docx [file pone.0274935.s003.docx]

# August, 2022

From: Authors

To: PLOS ONE editorials

**Subject: Response to reviewers**

Dear Editor and reviewers:

I would like to thank the editors and reviewers for their time, careful reading, and thorough and helpful remarks and questions about my manuscript, as well as the opportunity to improve and resubmit it.

I believe that my revised paper has substantially improved as a result of the reviewers’ remarks. I am glad to submit the revised research manuscript for consideration in PLOS ONE.

I responded to each reviewer's questions, comments, and suggested revisions one by one. I used the same letter to write my responses after copying the review decision from the editorial manager's submission menu. Editors' and reviewers' questions and comments are *italicized*, and my responses to the reviewers' comments are presented in plain text immediately below them. All of the modifications I made in the revised manuscript were referenced by page and line numbers. Please, follow my responses in a yellow mark to every comment or question in a green mark.

Once again, thank you for your time and attention to my paper***,*** and I eagerly await your response.

Sincerely!

Kenzudin Assfa Mossa (corresponding author)

Wolkite University

[kenzaheri@gmail.com](mailto:kenzaheri@gmail.com%20)

+251923702054

PONE-D-22-07638R1

Perceptions and knowledge of breast cancer and breast self-examination among young adult women in southwest Ethiopia: application of the health belief model

PLOS ONE

Dear Dr. Assfa,

Thank you for submitting your manuscript to PLOS ONE. After careful consideration, we feel that it has merit but does not fully meet PLOS ONE’s publication criteria as it currently stands. Therefore, we invite you to submit a revised version of the manuscript that addresses the points raised during the review process.

Please submit your revised manuscript by Sep 26 2022 11:59PM. If you will need more time than this to complete your revisions, please reply to this message or contact the journal office at plosone@plos.org. When you're ready to submit your revision, log on to https://www.editorialmanager.com/pone/ and select the 'Submissions Needing Revision' folder to locate your manuscript file.

Please include the following items when submitting your revised manuscript:

- A rebuttal letter that responds to each point raised by the academic editor and reviewer(s). You should upload this letter as a separate file labeled 'Response to Reviewers'
- A marked-up copy of your manuscript that highlights changes made to the original version. You should upload this as a separate file labeled 'Revised Manuscript with Track Changes'.
- An unmarked version of your revised paper without tracked changes. You should upload this as a separate file labeled 'Manuscript'.

If you would like to make changes to your financial disclosure, please include your updated statement in your cover letter. Guidelines for resubmitting your figure files are available below the reviewer comments at the end of this letter.

If applicable, we recommend that you deposit your laboratory protocols in protocols.io to enhance the reproducibility of your results. Protocols.io assigns your protocol its own identifier (DOI) so that it can be cited independently in the future. For instructions see: https://journals.plos.org/plosone/s/submission-guidelines#loc-laboratory-protocols. Additionally, PLOS ONE offers an option for publishing peer-reviewed Lab Protocol articles, which describe protocols hosted on protocols.io. Read more information on sharing protocols at https://plos.org/protocols?utm_medium=editorial-email&utm_source=authorletters&utm_campaign=protocols.

We look forward to receiving your revised manuscript.

Kind regards,

Alvaro Galli

Academic Editor

PLOS ONE

Journal Requirements:

Comments: *Please review your reference list to ensure that it is complete and correct. If you have cited papers that have been retracted, please include the rationale for doing so in the manuscript text, or remove these references and replace them with relevant current references. Any changes to the reference list should be mentioned in the rebuttal letter that accompanies your revised manuscript. If you need to cite a retracted article, indicate the article’s retracted status in the References list and also include a citation and full reference for the retraction notice.*

Response: I appreciate the suggestions. I checked the reference list; it is complete, correct, and free of any references that have been retracted. Scite (https://scite.ai/users/kenza-abazinab-pn5Ej/reference-checks) revealed no references that had been retracted or that had editorial concerns. In addition, I haven't changed the reference list.

[Note: HTML markup is below. Please do not edit.]

Reviewers' comments:

Reviewer's Responses to Questions

Comments to the Author

1. If the authors have adequately addressed your comments raised in a previous round of review and you feel that this manuscript is now acceptable for publication, you may indicate that here to bypass the “Comments to the Author” section, enter your conflict-of-interest statement in the “Confidential to Editor” section, and submit your "Accept" recommendation.

Reviewer #1: (No Response)

______________________________________

2. Is the manuscript technically sound, and do the data support the conclusions?

The manuscript must describe a technically sound piece of scientific research with data that supports the conclusions. Experiments must have been conducted rigorously, with appropriate controls, replication, and sample sizes. The conclusions must be drawn appropriately based on the data presented.

Reviewer #1: Yes

________________________________________

3. Has the statistical analysis been performed appropriately and rigorously?

Reviewer #1: Yes

________________________________________

4. Have the authors made all data underlying the findings in their manuscript fully available?

The PLOS Data policy requires authors to make all data underlying the findings described in their manuscript fully available without restriction, with rare exception (please refer to the Data Availability Statement in the manuscript PDF file). The data should be provided as part of the manuscript or its supporting information, or deposited to a public repository. For example, in addition to summary statistics, the data points behind means, medians and variance measures should be available. If there are restrictions on publicly sharing data—e.g. participant privacy or use of data from a third party—those must be specified.

Reviewer #1: Yes

________________________________________

5. Is the manuscript presented in an intelligible fashion and written in standard English?

PLOS ONE does not copyedit accepted manuscripts, so the language in submitted articles must be clear, correct, and unambiguous. Any typographical or grammatical errors should be corrected at revision, so please note any specific errors here.

Reviewer #1: Yes

________________________________________

6. Review Comments to the Author

Please use the space provided to explain your answers to the questions above. You may also include additional comments for the author, including concerns about dual publication, research ethics, or publication ethics. (Please upload your review as an attachment if it exceeds 20,000 characters)

*Reviewer #1: While most of the comment raised have been addressed in a few needs to be looked into.*

Abstract

Methods

Comments: *Line 24 which community Again, I suggest you State the community employed for the study.*

Response: I'm appreciative to the reviewer who made me aware of this. It has been updated and the new sentence read as: “A community-based cross-sectional study was carried out in Gurage zone, southwest Ethiopia, in 2021.”. The track changed document shows the modifications that have been made. Pages 2 and line 24 of the revised manuscript document contain the modifications.

Comments: *Line 48… replace occurred with occurs*

Response: I agree and the term occurred replaced by occurs. The changes made are indicated in track changed document. The changes are also found on the revised manuscript document [pages3, lines 49].

Comments: *Line 58.. again look at line 58.. the repetition of young women in the 2 sentences is not appropriate and should be modified.*

Response: Thank you for the insightful comments. The pronoun "they" was used in place of "young adult women" in the second sentence reads as: "They have a variety of issues, including a higher likelihood of biologically aggressive illness and metastatic disease at diagnosis, which results in a worse prognosis, more aggressive treatments and long-term treatment-related toxicities, and unique psychosocial concerns". The track changed document shows the modifications that have been made. Pages 3 and lines 59 of the revised manuscript document contain the modifications.

Comments: *Line 134… references for where questionnaire was adapted from, should be stated.*

Response: This is a valid observation. I have added references for where questionnaire was adapted from. The changes made are indicated in track changed document. The changes are also found on the revised manuscript document [pages7, lines 135-136].

Comments: *Line 139 use society… not society’s*

Response: I agree, and I've changed the word "society's" in the sentence with the word society. Pages 7 and line 140 of the revised manuscript document contain the modifications.

Comments: *Line 194 Again… the appropriate consent for participation should be a written informed consent not verbal agreement. This an important part of the study.*

Response: Thank you for sharing your thoughts. I have revised it. The new sentence read as follow: “The purpose of the study was communicated to the respondents, and signed consent from each participant was obtained prior to data collection." The changes made are indicated in track changed document. The changes are also found on the revised manuscript document [page10, lines 195-197].

Comments: *Line 216 and 217 … why do you have titles for tables and figures? Why present the same information in Tables and Figures. Explain please.*

Response: Your thoughtful concern is appreciated. The difference between the data shown in table 2 and fig. 1 is that the table pertains to the frequency distribution of responses given to each of the 11 items used to measure respondents' knowledge of breast cancer risk factors. In response to your suggestion, I eventually combined the data from Fig 1 into the table 2 and eliminated the Fig. The changes made are indicated in track changed document and found on the revised manuscript document [pages11, lines 215-217]. To overall knowledge of respondents about breast cancer risk factors categorized into three categories: low, moderate, and high. The frequency distribution is shown in the last three rows of table 2, page 12 of the revised manuscript.

Comments: *Line 228 should read…..* *Respondents knowledge about early warning signs of breast cancer, Gurage zone*

Response: Thank you so much for your comment, I have rephrased the title and the new sentence reads as: “Respondents knowledge about early warning signs of breast cancer, Gurage zone”. The changes made are indicated in track changed document. The changes are also found on the revised manuscript document [pages13, lines 229].

Comments: *In table 3… sd.. should be SD….. out of 11?explain please.*

Response: I agree with you, I have changed “sd” to “SD”. 11 is the sum score of the 11 items I used to generate the composite score of respondents' warning sign-related knowledge of breast cancer. The revised manuscript document (pages 13 and table 3) and the track changed document both indicate the changes that were made.

Comments: *Line 292 A stronger justification for difference is imperative.*

Response: thank you so much for valuable suggestions, I have added the possible factors that could contributed the reported difference in knowledge. the new sentences read as: “Since older and married women are more interested in and aware of BC (20,37,38), this difference could be explained by our younger participants (20–24 years of age), who were younger than those in Beirut (18–65 years), and whose proportion of married people (7.77%) was significantly lower than Beirut's (49.5%) (36)^”^. The changes made are indicated in track changed document and also found on the revised manuscript document [pages18, lines 292-296].

Comments: *Line 294 delete ..participated*

Response: I agree with your suggestion, and the term “participated” removed from the sentence. The changes made are indicated in track changed document.

Comments: *Line 354… rewrite as…..**only young adult women participated in the study.*

Response: Thank you for your comment, I have revised it and the new sentence reads as: “Since only young adult women participated in the study, it does not reflect the perceptions and knowledge of young adult males.” The changes made are indicated in track changed document. The changes are also found on the revised manuscript document [pages21, lines 358-359].

Comments: *Line 359… why threats?*

Response: I appreciate the query. It is well established that a slight or moderate threat plays a big part in making people worry about certain health issues, such as breast cancer. According to the findings of this study, which are shown in table 5, a significant number (96%) of survey participants were less concerned about breast cancer. Because of this it has been included in the conclusion.

________________________________________

7. PLOS authors have the option to publish the peer review history of their article (what does this mean?). If published, this will include your full peer review and any attached files.

If you choose “no”, your identity will remain anonymous but your review may still be made public.

Do you want your identity to be public for this peer review? For information about this choice, including consent withdrawal, please see our Privacy Policy.

Reviewer #1: No

________________________________________

[NOTE: If reviewer comments were submitted as an attachment file, they will be attached to this email and accessible via the submission site. Please log into your account, locate the manuscript record, and check for the action link "View Attachments". If this link does not appear, there are no attachment files.]

While revising your submission, please upload your figure files to the Preflight Analysis and Conversion Engine (PACE) digital diagnostic tool, https://pacev2.apexcovantage.com/. PACE helps ensure that figures meet PLOS requirements. To use PACE, you must first register as a user. Registration is free. Then, login and navigate to the UPLOAD tab, where you will find detailed instructions on how to use the tool. If you encounter any issues or have any questions when using PACE, please email PLOS at figures@plos.org. Please note that Supporting Information files do not need this step.

________________________________________

In compliance with data protection regulations, you may request that we remove your personal registration details at any time. (Remove my information/details). Please contact the publication office if you have any questions.

Thank you

The Author
